# Work Style Reform for Pediatric Residents

**DOI:** 10.31662/jmaj.2024-0419

**Published:** 2025-05-26

**Authors:** Hiro Nakao, Osamu Nomura, Naoya Tonegawa, Mitsuru Kubota, Akira Ishiguro

**Affiliations:** 1 Department of General Pediatrics and Interdisciplinary Medicine, National Center for Child Health and Development, Tokyo, Japan; 2 Center for Postgraduate Education and Training, National Center for Child Health and Development, Tokyo, Japan; 3 Medical Education Development Center, Gifu University, Gifu, Japan

**Keywords:** work style reform, resident, pediatric resident, wellness, burnout

## Abstract

**Introduction:**

Work style reform has affected pediatric residents’ balance between adequate training and wellness. This study aimed to investigate the impact of work style reform among pediatric trainees at the National Center for Child Health and Development (NCCHD), where several work style amendments were implemented from 2019 to 2024.

**Methods:**

We conducted a questionnaire-based cross-sectional survey of pediatric trainees in 2024 and compared the data with a previous survey from 2019 to evaluate the impact of work style reform. The questionnaire included demographic and work condition data, the Center for Epidemiologic Studies Depression Scale, and the Maslach Burnout Inventory (MBI).

**Results:**

Participants included 37 trainees (94.9%) in 2019 and 34 trainees (81.0%) in 2024. Median work hours per week (69.0-64.0, p = 0.04) and the frequency of night or holiday shift work (5-4 times/month, p = 0.002) decreased significantly in 2024. Compliance with daytime rest after night work also increased significantly (64.9%-91.2%, p = 0.01). All subscales of the MBI showed improvements, which remained significant after adjusting for demographics and work conditions (p < 0.001 for all).

**Conclusions:**

Work style reform has improved work conditions and wellness in pediatric training at NCCHD. Further reports from other medical specialties are awaited to assess the broader impact of work style reform on physicians.

## Introduction

Residency is a unique and important period for physicians that requires balancing adequate training with acceptable wellness. Pediatrics encompasses a vast range of children’s health, from neonatal to adolescent medicine, normal development to intensive care, and child care to guardian care. Therefore, the heavy workload and reduced wellness of pediatric residents are major concerns in Japan and worldwide ^(1), (2), (3), (4), (5)^. In Japan, the “Work Style Reform Bill” was partially enacted in 2019, and work style reform for physicians, which includes work hour regulations as a core component, was enforced in 2024 ^(6)^. Under this reform, overtime work is limited to 960 hours per year for the standard “A” level and 1,860 hours per year for the exceptional “B” or “C” levels. However, surveys in 2016-2022 reported that 20% to 40% of hospital physicians exceeded 960 overtime hours per year, whereas 4% to 10% exceeded 1,860 hours per year ^(6), (7)^. To comply with the reform and protect physicians’ wellness, hospitals have made necessary amendments to physician work environments ^(8), (9), (10), (11)^. However, we found limited information on work style reform for physicians, particularly in the pediatric field ^(12), (13), (14), (15), (16), (17)^.

The National Center for Child Health and Development (NCCHD) hosts the largest pediatric residency program in Japan and introduced an overnight call shift system in 2011―prior to the enactment of work style reform―to allow residents to have daytime rest before and after night shifts ^(4), (5)^. The short-term impact of this implementation was a reduction in work hours ^(5)^, while the long-term impact was improved mental wellness, which was associated with adherence to shiftwork system ^(4)^. From 2019 to 2024, the NCCHD residency program introduced further amendments to work style, the impact of which remains an area of interest given the scarcity of previous reports on work style reform for pediatricians.

In summary, this study aims to investigate the impact of work style reform on physicians in the NCCHD residency program.

## Materials and Methods

### Study setting

NCCHD is a 490-bed hospital specializing in child and perinatal care. It offers a 3-year residency program for pediatric residents to become pediatric specialists in accordance with the regulations of the Japan Pediatric Society, with 10-14 residents per training year. During their general pediatric ward rotation, which is the core content of their training, they participate in night shifts. Additionally, there are also 1-3 general pediatric fellows who have completed their 3-year residency and are training to become general pediatric specialists.

### Work style reform

Following the work style reform for physicians ^(6)^, several amendments have been implemented in the general ward rotation of the pediatric residency program at NCCHD between 2019 and 2024.

First, efforts have been made to substantially reduce working hours. All general pediatric teams have moved afternoon team conferences and medical rounds, previously held around 6-7 p.m., to before 4 p.m. during regular working hours. Additionally, other departments and medical professionals were asked to hold multidisciplinary conferences during working hours.

Second, the group physician-in-charge system has been fully incorporated. Under this system, at least 3 physicians―a primary physician, a secondary physician, and a supervising physician―are assigned to each patient. This ensures that when the primary physician is absent, a substitute physician can provide care.

Third, measures have been implemented to ensure compensatory duty-off days for holiday shift work, as introduced in the work style reform. Each team now prepares a monthly work shift schedule to enable all trainees to take their compensatory duty-off days. The group physician-in-charge system has also contributed to making these duty-off days feasible for trainees.

### Design and participants

We conducted a questionnaire-based cross-sectional survey of 42 pediatric trainees enrolled in 2024 and compared the data to a previous 2019 study of 39 trainees ^(4)^. The data were collected online and anonymously using the Microsoft Forms^Ⓡ^ platform. To minimize reporting bias and eliminate malicious responses, we reviewed each dataset individually to check for missing or unnatural entries.

### Data collection

We collected the following demographic data: age, sex, marital status, year of residence, and post-graduate year. Work conditions data included weekly working hours, the number of night or holiday duty shiftwork events per month, the number of off-duty days per month, and compliance with the overnight call shift system, defined as having daytime off before or after night work. To assess mental wellness, we used the Center of Epidemiologic Studies Depression Scale (CES-D) and Maslach Burnout Inventory (MBI), both widely used for screening depression and burnout, respectively. The CES-D cutoff is 16 points, with scores of 16 and above indicating depressive symptoms. The MBI consists of 3 components: Emotional Exhaustion (EE), Depersonalization (DP), and Personal Accomplishment (PA).

### Analysis

We expressed categorical variables as percentages (numbers) and continuous variables as median (interquartile ranges), with some also presented as mean ± standard deviations. We compared working conditions data and mental wellness scores between 2019 and 2024, when the “Work Style Reform Bill” was enacted and work style reform for physicians was enforced, respectively ^(6)^. We used the Mann-Whitney *U* test for continuous variables and Fisher’s exact test for categorical variables, with a p < 0.05 considered statistically significant. We also conducted multivariable regression analyses with mental wellness scores, including CES-D and MBI subscales, as outcome variables for 2019 and 2024 participants. Explanatory variables included demographic and work condition data, with multicollinearity taken into account. We performed the analysis using EZR (version 1.65) statistical software, which is based on R and R commander.

### Ethical considerations

The study data were anonymized and approved by the Ethics Committee of NCCHD (number: 2318). Online consent was obtained from all survey participants. This paper was written in accordance with the Strengthening the Reporting of Observational Studies in Epidemiology reporting guidelines.

## Results

**Figure 1.**
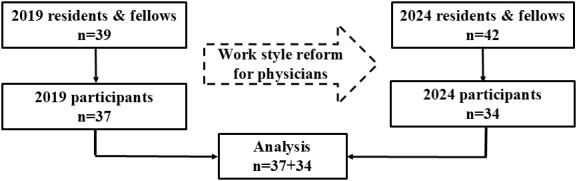
Study flow chart. Eligible pediatric residents and general pediatric fellows numbered 39 and 42 in 2019 and 2024, respectively. Of these, 37 and 34 participated in the cross-sectional study each year. We compared and analyzed the data from 2019 and 2024, corresponding to the enactment of “Work Style Reform Bill” and the enforcement of the work style reform for doctors, respectively.

The response rate for the current 2024 survey was 81.0% (34 participants/42 enrolled). We compared the current data with data from the previous 2019 survey (response rate: 94.9%; 37/39) ^(4)^, which was conducted when the “Work Style Reform Bill” began to be enacted (Figure 1). There was no missing data for any participants.

### Demographics and work conditions

Demographics and work conditions data in 2019 and 2024 are listed in Table 1. From 2019 to 2024, both work hours (median: from 69.0 to 64.0 hours/week, p = 0.04) and the frequency of night or holiday shiftwork (median: from 5 to 4 times/month, p = 0.002; mean: from 4.8 to 4.0 times/months) decreased significantly. The percentage of those who could take off during the day after night shifts also increased significantly in 2024 (from 64.9% to 91.2%, p = 0.01). However, there were no statistically significant changes from 2019 to 2024 in the number of off-duty days (median: from 4 to 4 days/month, p = 0.07; mean: from 4.1 to 4.5 days/months) or in the percentage of compliance with daytime rest before night shifts (from 70.3% to 85.3%, p = 0.16).

**Table 1.** Demographics and Work Conditions Data.

		2019 (n = 37)	2024 (n = 34)	p-Value
**Age, years**†		29 (28-31)	28 (27-30)	NA
**Male**‡		45.9 (17)	44.1 (15)	NA
**Married**‡		29.7 (11)	29.4 (10)	NA
**1st year resident**‡		32.4 (12)	32.4 (11)	NA
**2nd year resident**‡		27.0 (10)	29.4 (10)	NA
**3rd year resident**‡		29.7 (11)	32.4 (11)	NA
**PGY, years**†		4 (3 to 5)	4 (3 to 5)	NA
**Work time, hours/week**†		69.0 (63.0-72.0)	64.0 (60.0-69.0)	0.04§
**Night or holiday shiftwork, ** **times/month**††		5 (4-5)	4 (4-5)	0.002§
		4.8 ± 0.9	4.0 ± 1.2	
**Off-duty days, days/month**††		4 (3-5)	4 (4-5)	0.07§
		4.1 ± 1.1	4.5 ± 0.9	
**Compliance with daytime off**	**before nightwork**‡	70.3 (26)	85.3 (29)	0.16¶
	**after nightwork**‡	64.9 (24)	91.2 (31)	0.01¶

† represents median (interquartile range). ‡ represents % (number). †† represents median (interquartile range) and mean ± standard deviation. § represents Mann-Whitney *U* test. ¶ represents Fisher’s exact test. PGY: post-graduate years; NA: not applicable.

### Mental wellness

Table 2 describes the CES-D and MBI data from 2019 and 2024. Higher CES-D and MBI scores indicate poorer mental wellness, specifically greater depressive or burnout tendencies. The percentage of residents with depressive symptoms (CES-D ≥ 16) showed no improvements from 2019 to 2024 (from 21.6 to 20.6, p = 1.00), and the reduction in the median CES-D score (from 10.0 to 7.5, p = 0.55) was not statistically significant. However, the median scores of all MBI subscales significantly decreased from 2019 to 2024 (EE: from 12.0 to 7.0, p < 0.001; DP: from 9.0 to 2.0, p < 0.001; PA: from 17.0 to 12.0, p < 0.001).

**Table 2.** Mental Wellness and Patient Care Ownership.

	2019 (n = 37)	2024 (n = 34)	p-Value
**CES-D**
**Residents with CES-D ≥ 16**†	21.6 (8)	20.6 (7)	0.55§
**CES-D score**‡	10.0 (5.0-14.0)	7.5 (5.3-13.0)	0.55¶
**MBI**			
**Emotional exhaustion**‡	12.0 (10.0-14.0)	7.0 (4.0-10.0)	<0.001¶
**Depersonalization**‡	9.0 (8.0-10.0)	2.0 (1.3-5.0)	<0.001¶
**Personal accomplishment**‡	17.0 (14.0-19.0)	12.0 (9.3-15.0)	<0.001¶

CES-D: Center for Epidemiologic Studies Depression scale; MBI: Maslach Burnout Inventory Higher scores of CES-D or all subscales of MBI mean more depressive or burnout tendencies, respectively. † represents % (number). ‡ represents median (interquartile range). § represents Fisher’s exact test. ¶ represents Mann-Whitney *U* test

Multiple regression analyses indicated that the survey year was significantly associated with all MBI subscales (β: EE 4.7, DP 5.9, PA 4.8; all p <0.001), in other words, MBI improvements in 2024 remained significant even after adjusting for demographic and work condition variables (Table 3). On the other hand, no demographic or working condition variables were significantly associated with mental wellness scores in these regression analyses.

**Table 3.** Multivariable Regression Analyses with Mental Wellness Scales as Outcome Variables for Participants in 2019 and 2024 (n = 71).

Outcome variable	CES-D		MBI-Emotional exhaustion	
Explanatory variable	β (95% CI)	p-Value	β (95% CI)	p-Value
**Investigation year**†	−0.08 (−3.8 to 3.5)	0.97	4.7 (2.7 to 6.7)	<0.001
**Age**	0.4 (−0.6 to 1.4)	0.47	0.3 (−0.3 to 0.9)	0.29
**Sex**‡	1.1 (−2.3 to 4.5)	0.52	−0.9 (−2.7 to 1.0)	0.36
**Married**§	−1.5 (−5.0 to 2.0)	0.39	0.7 (−1.2 to 2.6)	0.48
**Resident year**	0.5 (−1.7 to 2.7)	0.66	0.5 (−0.7 to 1.7)	0.42
**Work time**	0.16 (−0.1 to 0.4)	0.15	0.1 (0.0 to 0.2)	0.06
**Night or holiday shiftwork**	0.1 (−1.5 to 1.7)	0.89	0.5 (−0.4 to 1.4)	0.25
**Off-duty days**	1.6 (−0.1 to 3.2)	0.06	0.8 (−0.1 to 1.7)	0.09
**Daytime off before night work**¶	−1.8 (−6.3 to 2.7)	0.43	−0.6 (−3.1 to 1.8)	0.61
**Daytime off after nightwork**¶	0.0 (−4.5 to 4.5)	1.00	2.0 (−0.4 to 4.5)	0.10
**Outcome variable**	**MBI-Depersonalization**		**MBI-Personal accomplishment**	
**Explanatory variable**	**β (95% CI)**	**p-Value**	**β (95% CI)**	**p-Value**
**Investigation year**†	5.9 (43 to 7.5)	<0.001	4.8 (2.3 to 7.2)	<0.001
**Age**	0.2 (−0.2 to 0.7)	0.28	−0.2 (−1.0 to 0.5)	0.49
**Sex**‡	0.1 (−1.4 to 1.6)	0.89	0.2 (−2.2 to 2.5)	0.89
**Married**§	−0.1 (−1.6 to 1.4)	0.90	0.3 (−2.0 to 2.7)	0.77
**Resident year**	0.3 (−0.7 to 1.3)	0.51	0.3 (−1.2 to 1.9)	0.66
**Work time**	0.0 (−0.1 to 0.1)	0.39	0.0 (−0.2 to 0.1)	0.66
**Night or holiday shiftwork**	0.2 (−0.5 to 0.9)	0.57	−0.3 (−1.4 to 0.8)	0.62
**Off-duty days**	0.6 (−0.1 to 1.4)	0.09	−0.4 (−1.5 to 0.8)	0.50
**Daytime off before night work**¶	0.8 (−1.2 to 2.7)	0.45	−1.8 (−4.8 to 1.3)	0.25
**Daytime off after nightwork**¶	0.8 (−1.2 to 2.7)	0.44	0.1 (−3.0 to 3.1)	0.97

CES-D: Center for Epidemiologic Studies Depression scale; CI: confidence interval; MBI: Maslach Burnout Inventory; β: partial regression coefficient. Dummy variables were labeled as follows: † 0: 2024, 1: 2019; ‡ 0: female, 1: male; § 0: not married, 1: married; ¶ 0: not compliant, 1: compliant.

## Discussion

The current study investigated changes in work conditions and the mental wellness of pediatric trainees at a children’s hospital between 2019, when the “Work Style Reform Bill” was enacted, and 2024, when work style reform for physicians was enforced ^(6)^. With the implementation of several work style amendments, both work time and night and holiday duty shiftwork have decreased, and compliance with daytime rest after night work has improved. The burnout score also improved in 2024.

The demonstrated improvements in work conditions could be related to our implementation of work style amendments. Efforts to reduce work time have likely been successful. The group physician-in-charge system and compensatory duty-off days may also have contributed to reducing work hours and ensuring compliance with the shiftwork system. On the other hand, it remains unclear why night and holiday duty shiftwork has decreased based solely on our amendments. Other factors, such as changes in residency rotations or adjustments to monthly work shifts during this period, may have played a role. This remains an area for further research. Additionally, further efforts are required to reduce work time, as the median weekly work time in the current study was 64 hours, equating to 24 hours of overtime, assuming a standard 40-hour workweek. This overtime exceeded the “A” level of work style reform for physicians, which defines normal overtime for physicians as 960 hours per year (approximately 20 hours per week) ^(6)^.

The current regression analyses indicated that the burnout tendency of trainees improved in 2024, even after adjusting for demographic and working condition data. However, work time, shiftwork frequency, and compliance with the shiftwork system were not associated with improvements in the MBI scores in the regression analyses, despite improvements in these factors. In our previous research, shiftwork compliance was detected to be related to good mental wellness ^(4)^. This raises another question: What factors, other than work conditions, could improve the mental wellness of trainees? Other reported factors influencing residents’ wellness include exercise, stress reduction, autonomy, competence, social relatedness, sleep, learning conditions, and appropriate support ^(2), (3), (18), (19), (20), (21), (22), (23), (24), (25), (26)^. Qualitative research may help identify additional factors that contribute to improved wellness in our training environment.

Potential negative impacts of work style reform for trainees include increased burdens on faculty members, who may need to compensate for the reduction in trainees’ workload, and a decline in medical knowledge or skills due to reduced training hours. In addition, concerns have been raised about a decrease in patient care ownership, a sentiment shared by both residents and faculty at the NCCHD in the context of reduced training hours (a report in Japanese). The concept of patient care ownership, which is still being defined, refers to a comprehensive sense of responsibility for the care of each patient. It has gained worldwide attention since the 2010s, following work-time regulation initiatives for trainees ^(27), (28), (29), (30), (31), (32), (33), (34), (35)^. Its scale has become more widely available recently ^(36), (37), (38)^ and could serve as a baseline score in our 2024 survey (data not shown). We will continue researching to develop a training environment that enhances patient care ownership, as well as medical knowledge and skills, while promoting trainees’ wellness.

The current study design was a repeated cross-sectional study, not a cohort study. Thus, the compared groups of trainees may have had different characteristics, although there was no change in the trainee selection process or demographic data (Table 1) between 2019 and 2024. In addition, this design helped avoid response bias by repeating the same questionnaire for the same group. Another limitation is the generalizability of the findings, as the current study only enrolled pediatric trainees. There may be differences in working climates and training environments in other medical specialties, such as surgical or perinatal departments. This survey provides a model for work style reform in pediatrics, and further reports from other specialties are anticipated to expand the scope of medical training and work styles.

### Conclusions

Work style reform for physicians has positively progressed in pediatric training, with improved work conditions and a reduced tendency for burnout. Further reports are awaited to provide additional insights into the impact of work style reform across various medical specialties.

## Article Information

### Conflicts of Interest

None

### Sources of Funding

This study was supported by grants from the National Center for Child Health and Development in Japan (2023E-1 and 2024C-13).

### Acknowledgement

We thank all the pediatric residents and fellows who participated in this study, as well as the senior medical English editor at the Center for Postgraduate Education and Training at NCCHD for editing this article.

### Author Contributions

Hiro Nakao, Osamu Nomura, and Akira Ishiguro conceived the ideas; Hiro Nakao, Naoya Tonegawa, and Akira Ishiguro curated the data; Hiro Nakao and Osamu Nomura analyzed the data; Naoya Tonegawa, Mitsuru Kubota, and Akira Ishiguro supervised the investigation; Hiro Nakao and Osamu Nomura wrote the original draft; Naoya Tonegawa, Mitsuru Kubota, and Akira Ishiguro reviewed and edited the draft; and all authors approved the final manuscript.

### Approval by Institutional Review Board (IRB)

This study was approved by the Ethics Committee of the National Center for Child Health and Development (number: 2318).

### Data Availability Statement

The data supporting this study’s findings are available from the corresponding author upon reasonable request.
